# A Case of Histiocytic Sarcoma Arising from Mycosis Fungoides

**DOI:** 10.1155/2019/7834728

**Published:** 2019-10-03

**Authors:** Ethan A. Burns, Cesar Gentille, Saro Kasparian, Sai Ravi Pingali

**Affiliations:** ^1^Houston Methodist Hospital, Department of Medicine, 6550 Fannin St, Ste 1101, Houston, TX 77030, USA; ^2^Houston Methodist Cancer Center, 6445 Main St Floor 24, Houston, TX 77030, USA

## Abstract

Histiocytic sarcoma (HS) is an uncommon malignant neoplasm arising from mature histiocytes and most commonly characterized by the immunophenotypic expression of CD68, CD163, or lysozyme. Although rare, HS arising as a second primary malignancy following hematolymphoid neoplasms has been reported. To our knowledge, this is the first reported case of HS occurring as a second primary malignancy in a patient with mycosis fungoides (MF), with the retained immunophenotype markers CD30 and CD4.

## 1. Introduction

The hematopoietic system is comprised of distinct cellular lineages uniquely delineated by morphologic, immunophenotypic, and functional characteristics [1, 2]. These cells are considered lineage committed once they are fully differentiated [2], which is defined by the expression of specific genes and phenotypic markers. However, under certain circumstances, fully differentiated cells may inherit mutations making it possible to undergo a transformation into a distinctly new cell line [2]. A rare, but well-documented example is HS arising as a secondary or concurrent malignancy in association with hematologic malignancies. This is the first known report of HS arising in a patient with previously diagnosed MF.

## 2. Case

The patient is a 61-year-old African American female with a long history of MF stage IB that first presented with symptoms of pruritic cutaneous lesions on her extremities at the age of 10 years, though the diagnosis of MF was not confirmed via skin biopsy until the age of 36 years. She had a long treatment course, but her disease was refractory to multiple modalities including psoralens and long-wave ultraviolet radiation, interferon, isotretinoin, nitrogen mustard, total body skin beam radiation, bexarotene, and acitretin. Two years prior to her admission, she developed a rapidly enlarging left hip tumor. Biopsies confirmed MF without large-cell transformation that was positive for CD4 and CD30. She received localized external beam radiation for 5 treatments (2,000 cGY). Following radiation therapy, she developed an ulcer in the tumor bed. A punch biopsy indicated chronic inflammation and scar but no evidence of tumor recurrence.

**Figure 1:**
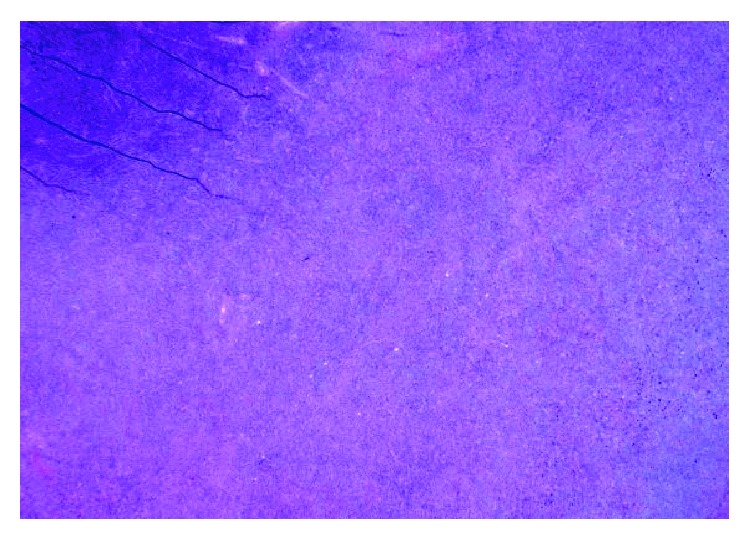
Low-power view of the left inguinal lymph node biopsy demonstrating complete effacement of normal lymph node architecture.

**Figure 2:**
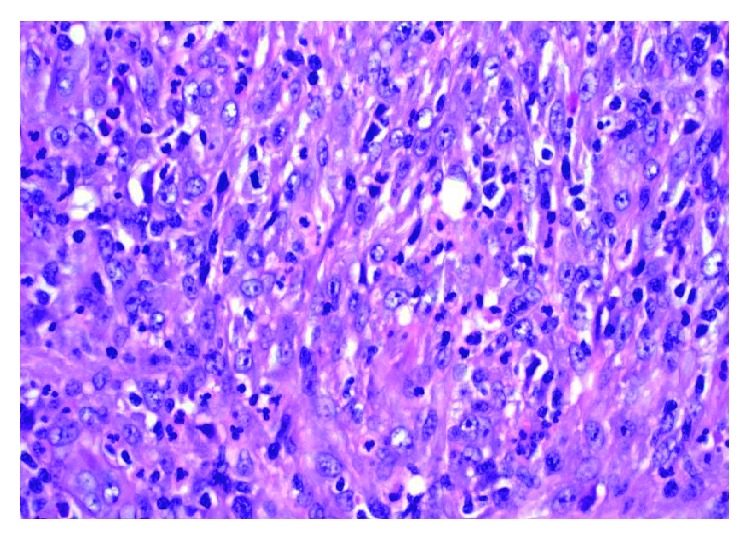
Effaced inguinal lymph node with spindled and epithelioid cells with eosinophilic cytoplasm. Large pleomorphic nuclei with abundant nucleoli.

**Figure 3:**
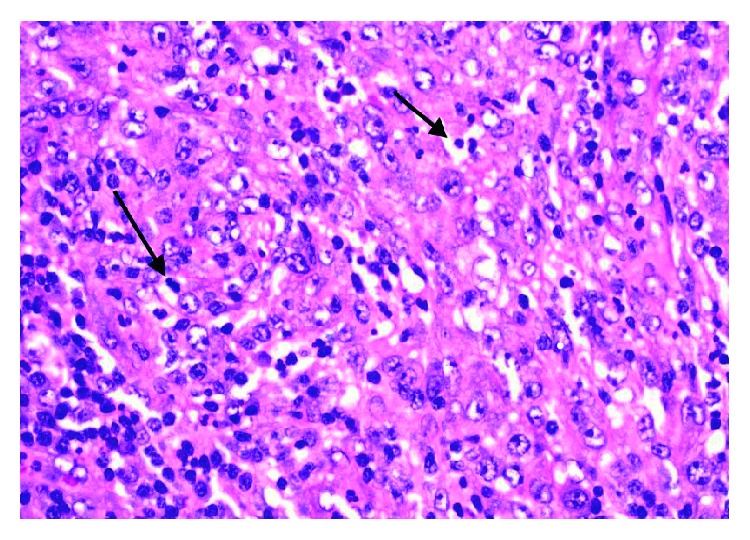
Effaced inguinal lymph node with frequent mitoses and apoptosis.

**Figure 4:**
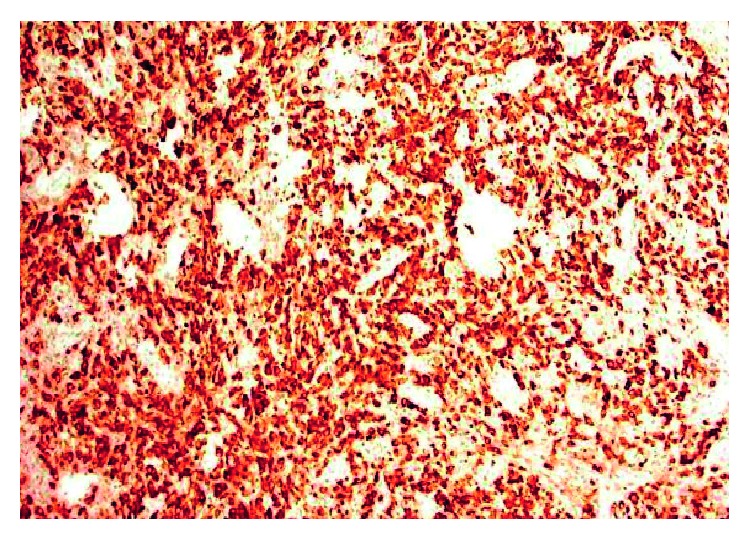
Inguinal lymph node CD30+ stain.

**Figure 5:**
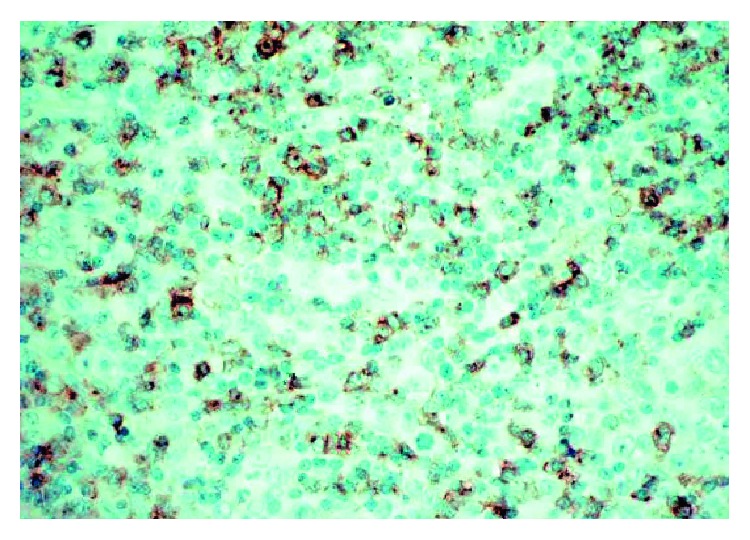
Inguinal lymph node CD4+ stain.

**Figure 6:**
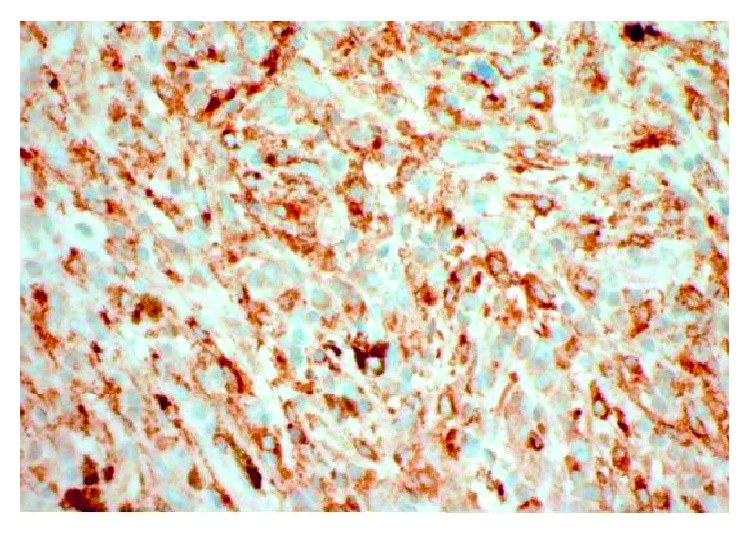
Inguinal lymph node CD163+ stain.

**Figure 7:**
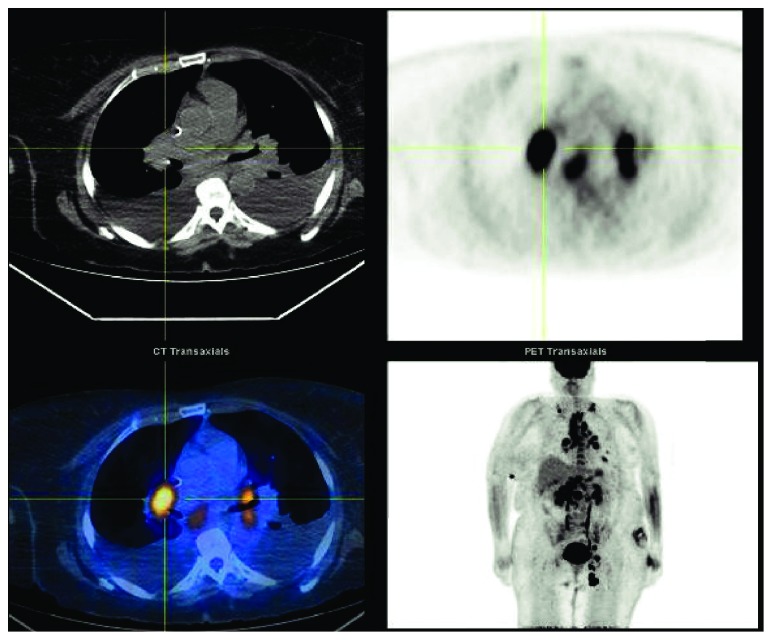
Positron emission tomography (PET) scan assessment of tumor burden before CHOEP chemotherapy initiation. Increased uptake in the bilateral supraclavicular (SUV 10.9), paratracheal (SUV 12.1), hilar (SUV 12.9), periportal (SUV 10.3), left inguinal (SUV 16.9) lymph nodes, pulmonary nodule (SUV 6.1), and right hepatic lobe (SUV 4.7). CHOEP: cyclophosphamide, Adriamycin, vincristine, etoposide, and prednisone.

Several months after the biopsy, the patient developed weakness, fatigue, fever, and increasing serosanguinous discharge from her left hip wound and was admitted to the hospital for sepsis. Computed tomography (CT) scans were significant for diffuse diaphragmatic, abdominal, pelvic, and inguinal lymphadenopathy concerning for lymphoma, suspected to be metastatic visceral spread from her underlying MF. She had an excisional left inguinal lymph node biopsy which revealed an enlarged lymph node with effaced architecture due to diffuse proliferation of neoplastic spindle cells (Figures 1–6). Positive tumor markers included CD4, CD30, CD45, CD43, CD68, and CD163, with a 37% MIB-1 proliferative index, consistent with HS (Figures 1–6). Positron emission tomography (PET) imaging indicated widely metastatic disease, with increased uptake in numerous lymph nodes, pulmonary nodules, and the liver (Figure 7). She had a bone marrow biopsy without a clonal B-cell population, aberrant T-cell markers, or immunophenotypic myeloblasts. She was started on cyclophosphamide, adriamycin, vincristine, prednisone, and etoposide (CHOEP) and was discharged with follow-up.

**Figure 8:**
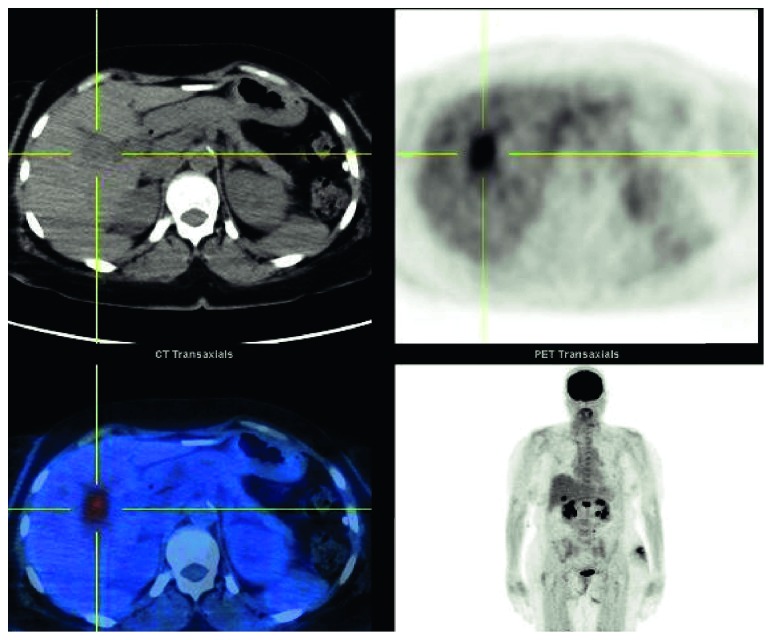
Follow-up PET after the 3^rd^ cycle of CHOEP. Interval resolution of supraclavicular, hilar, and axillary uptake. Increased uptake in the right hepatic lobe (SUV 5.8). New uptake in L1 (SUV 8.8). PET: positron emission tomography; CHOEP: cyclophosphamide, Adriamycin, vincristine, etoposide, and prednisone.

**Figure 9:**
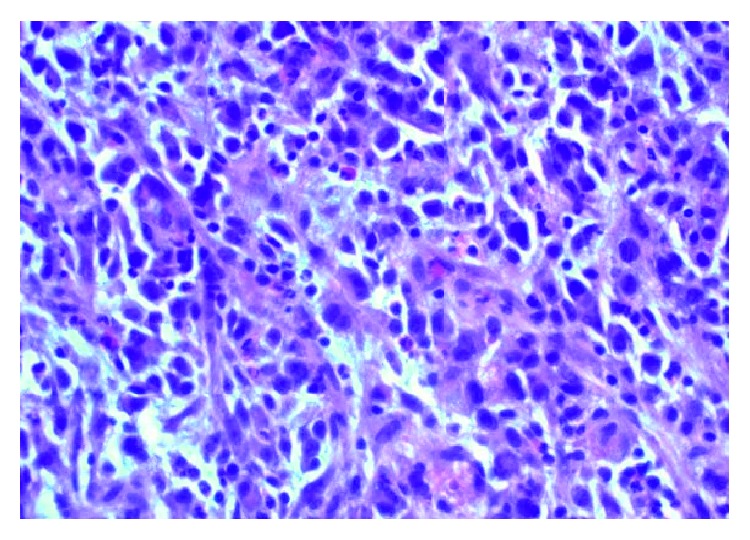
Liver parenchyma infiltrated with large spindle cells with abundant foamy clear to eosinophilic cytoplasm and large pleomorphic nuclei with abundant nucleoli. Mixed inflammatory infiltrates, consisting of neutrophils, eosinophils, lymphocytes, and plasma cells.

The patient had a repeat PET scan after completion of her third cycle of CHOEP that demonstrated a new lesion in the first lumbar vertebrae and increased uptake in her liver concerning for disease progression (Figure 8). She had a biopsy of the liver mass lesion which was identical to the HS noted on the previous excisional lymph node biopsy (Figures 2–9). Prior to resuming her chemotherapy, the patient developed nausea, vomiting, and abdominal pain and was admitted again. She had elevated alkaline phosphatase, bilirubin, and right upper quadrant pain concerning for acute cholangitis. Imaging revealed a large intrahepatic mass and extensive intra-abdominal and mediastinal lymphadenopathy. Due to her treatment-resistant disease, she was started on cladribine and cytarabine. Her hospital course was complicated by acute hypoxemic respiratory failure and ascending cholangitis requiring a delay in chemotherapy. She further progressed to multiorgan failure, so her family decided to proceed with comfort care measures.

## 3. Discussion

HS is an uncommon malignancy that arises from mature macrophages and diagnosed by the molecular expression of CD68, CD163, or lysozyme (Table 1) [3]. Clinically, it is associated with the typical “B” symptoms seen in non-Hodgkin lymphoma, including fevers, night sweats, and weight loss, and may also present with cytopenias, lymphadenopathy, or hepatosplenomegaly. The incidence is 0.17 per 1,000,000 individuals, occurs more frequently in Caucasian males, and has a median age of 63 years at diagnosis [4]. The most common initial sites of involvement include the skin and connective tissues (35.8%), lymph nodes (17%), respiratory tract (8.2%), and central nervous system (7.5%) [4]. Staging workup, including imaging studies such as CT or PET/CT, is helpful in determining the extent of disease spread, but excisional biopsy with tissue confirmation is necessary to confirm the diagnosis [5]. There is no standardized treatment algorithm for this aggressive malignancy. The overall median survival is 6 months, which appears to be independent of the primary site or ethnicity [4].

There is a clear, albeit rare association between HS and hematolymphoid malignancies via retained genetic and molecular abnormalities [5]. HS arising as a secondary malignancy occurs in approximately 21% of HS cases and is most often reported following hematologic malignancies arising from B-cell origin [4, 5]. The 2008 World Health Organization revised the diagnostic criteria for HS to include immunoglobulin heavy chain and T-cell receptor rearrangements that were classified as transdifferentiated forms of various other hematolymphoid malignancies [6].

**Figure 10:**
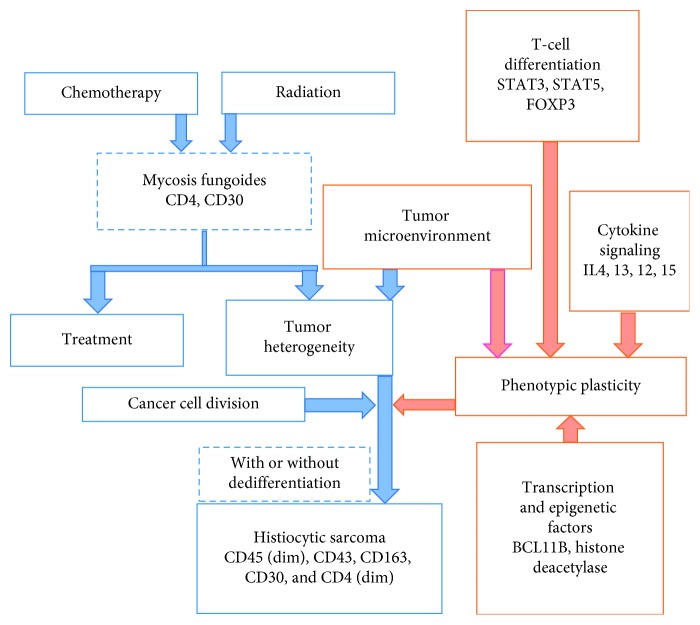
Theoretical malignant evolution and factors contributing to second primary HS in a patient with a protracted history of mycosis fungoides.

The mechanism of HS transformation from hematolymphoid malignancies remains poorly understood and controversial. Transformation is believed to occur via three mechanisms. Transdifferentiation is a well-studied and reported process in malignancies arising from B cells, occurring when lineage-committed B cells convert into phenotypically distinct cells with similar genetic makeup [5]. The second theorized mechanism is a two-step process via dedifferentiation to a common precursor cell followed by transdifferentiation to a different cell line. Finally, transdetermination involves differentiation of a lineage committed but not fully differentiated cell into another [5, 7]. The latter has been reported in T-cell lymphoma but occurred concomitantly with two other malignancies [7]. This is the first reported case of HS arising in a patient with MF, interestingly, with retention of immunophenotypic markers of the initial malignancy. In this case, genetic tests were not conducted on the skin biopsies diagnostic of MF or on the lymph node biopsy diagnostic of HS, so a common genetic mutation definitively linking the two malignancies and thus transformation could not be established. Typically, when HS arises from a lymphoid neoplasm it has a distinctly unique immunophenotype with retained genetic mutations. However, in this case, there was evidence of retained immunophenotype markers CD30 and CD4, unique findings that introduces the possibility of a malignant transformation between the two malignancies. The mechanism by which the secondary HS arose following decades of treatment-resistant MF is not defined, but is likely to have occurred via a myriad of factors, including a combination of transcription factors and epigenetic modifications, a dynamic microenvironment resulting in severe immunosuppression [8], alteration in cytokine receptor signaling, and selection pressure (Figure 10).

In MF, disease pathogenesis is believed to stem from apoptotic resistance by the malignant monoclonal T-cell infiltrate, which has been linked to aberrant overexpression of the BCL11B transcription factor [9, 10]. Further augmenting this mechanism, cutaneous T-cell lymphoma (CTCL) cells have demonstrated resistance to FAS-mediated apoptosis and growth suppression by TGF-*β* [11]. With advancing tumor and disease stage in CTCL, BCL11B activity appears to increase, indicating a potential role in disease progression [9]. Moreover, BCL11B interacts with histone deacetylases, which are functional classes of epigenetic protein complexes involved in various arrays of cellular functions including regulation of gene expression and tumorigenesis [10, 12]. As MF advances, clonal dominance of the TH2 cytokines IL4 and IL13 predominate allowing for clonal proliferation [13], immune dysregulation, and subsequent disease progression [11, 14]. It is possible that over decades, alteration in this patient's aberrant transcription factors and epigenetic regulator modifications allowed for mutations that permitted cellular transformation.

T-cell lymphomas are a heterogenous group of lymphoproliferative malignancies, and the subgroup that comprises CTCL has a dynamic and alternating microenvironment with a high phenotypic plasticity. In general, phenotypic plasticity is due to a host of factors that work to evade antitumor immunity and ultimately results in severe immunosuppression [8]. In addition to genetic and epigenetic modifications, the tumor microenvironment plays a pivotal role in malignant heterogeneity [8]. STAT3 and STAT5 are important mediators of plasticity and important determinants of T-cell differentiation in CTCL. These serve to weaken antitumor immunity by promoting the differentiation to TH17 and Treg cells [15]. The cytokines IL-2 and IL-15 play a role in the stimulation of malignant T-cell phenotype through JAK3/JAK3 kinase phosphorylation cascades as well as IL-10 and FOXP3 expression [8]. Overtime, these factors culminate in severe immunosuppression, which appears to be due to disruption of the T-cell repertoire [16]. It is possible that hyperactivity of these factors impacting tumor heterogeneity and plasticity, coupled with tumor treatment resistance and selection pressure created an ideal environment that allowed for class switching to HS (Figure 10).

Cases of HS arising as a secondary malignancy directly attributed to selection pressure are lacking, so it is not known if this plays a direct or contributory role in malignant transformation. However, hematolymphoid malignancies and specifically CTCL are known to have tumor heterogeneity, so it is possible they are predisposed to treatment-driven selection pressure. Genetic heterogeneity may lead to subpopulations of treatment-sensitive and treatment-resistant clones, a phenomenon seen in various malignancies. Upon treatment initiation, the elimination of treatment-sensitive clones allows for “competitive release,” or the minimalization of competitive forces of the treatment-sensitive dominant clone on the treatment-resistant subclone [17]. This allows for treatment-resistant subclones to repopulate and drive the tumor, which may be genetically distinct and carry more genetic abnormalities than the initial treatment-sensitive clone [18, 19]. For example, Tang et al. suggested that treatment with imatinib in chronic myeloid leukemia (CML) diminished CML clones with the most aggressive malignant potential [20]. However, CML clones have dichotomous kinetics that may allow more slowly dividing leukemic cell lines to relapse after discontinuation of imatinib [20]. In addition to “competitive release,” chemotherapy-induced mutagenesis can further drive malignant evolution [17].

The carcinogenic potential of radiation therapy is well known and attributed to approximately 5% of second primary malignancies [21]. Ionizing radiation can precipitate breaks in double-stranded DNA and alteration in DNA repair, which may lead to malignant transformation of the irradiated cell and increase the risk of secondary malignancies [21, 22]. Radiation-induced sarcomas are the second most common malignancy that can arise following radiation therapy, and typically arise 10–12 years following radiation therapy, with an arbitrary cutoff period of 3–4 years to delineate sporadic sarcomas [23, 24]. There is an isolated case report of a central nervous system HS arising following radiation therapy (CD163, CD68, and CD4 positive) [25], and it is known that radiation therapy in patients with non-Hodgkin's lymphoma are at increased risk for second malignancies including sarcomas [26], so it is possible that radiation played a role in the formation of this second primary malignancy. The patient in this case had treatment-resistant and recurrent MF for five decades. Prior to radiation therapy, biopsy of her right hip tumor was positive for CD4 and CD30 without large-cell transformation. While selection pressure alone cannot be implicated as the primary cause of malignant transformation, it may have been one of a complex array of pathogenic mechanisms leading to this malignant transformation.

## 4. Conclusion

This is the first presentation of HS arising as a secondary malignancy from a patient with MF, supported by the retention of CD4 and CD30 immunophenotype from the primary cancer. Although uncommon, hematolymphoid malignancies of T-cell origin, including cutaneous T-cell lymphomas, may precipitate a secondary HS. This concerning and often fatal second malignancy should be considered in patients with ongoing or previous management of a hematolymphoid malignancy presenting with new or rapid disease progression.

## Figures and Tables

**Table 1:** A comparison of the immunophenotypic, cytomorphologic, and clinical findings of HS and MF.

Malignancy	Histiocytic sarcoma	Mycosis fungoides
Immunophenotype	CD68	CD4
	CD163	CD8
	Lysozyme	CD56
	May be variable	CD4/8-CD30^¥^
Cytomorphology	Large and round oval nuclei with spindling; large, atypical pleomorphic lymphocytes with eosinophilic cytoplasm	T cells in epidermis and dermis with ceribriform nuclei; Pautrier micro abscesses in epidermis
	May be variable	
Clinical findings	Unifocal to metastatic disease with systemic symptoms; preferentially involves skin, soft tissue, or the gastrointestinal tract	Cutaneous patches/plaques or tumors that may be localized or diffuse

^¥^CD30 (Ki-1) positivity may be seen in primary cutaneous anaplastic large-cell lymphoma, lymphomatoid papulosis, pagetoid reticulosis, and transformed MF.

## Abbreviations

HS: Histiocytic sarcoma
MF: Mycosis fungoides
CT: Computed tomography
PET: Positron emission tomography
CHOEP: Cyclophosphamide, adriamycin, vincristine, etoposide, and prednisone
CTCL: Cutaneous T-cell lymphoma
CML: Chronic myeloid leukemia.
